# Contemporary Impact of circadian symptom-onset patterns of acute ST-Segment elevation myocardial infarction on long-term outcomes after primary percutaneous coronary intervention

**DOI:** 10.1080/07853890.2020.1863457

**Published:** 2020-12-21

**Authors:** Hui Peng, Zhijun Sun, Beibing Di, Xiaosong Ding, Hui Chen, Hongwei Li

**Affiliations:** a Department of Cardiology, Cardiovascular Center, Beijing Friendship Hospital, Capital Medical University, Beijing, P. R. China; b Department of Internal Medical, Medical Health Center, Beijing Friendship Hospital, Capital Medical University, Beijing, P.R. China; c Beijing Key Laboratory of Metabolic Disorder Related Cardiovascular Disease, Beijing, P. R. China

**Keywords:** ST-segment elevation myocardial infarction, Circadian rhythm, Major adverse cardiovascular event, Reperfusion

## Abstract

**Background:**

Daytime variation with regard to onset time of ST-elevation myocardial infarction (STEMI) symptoms has been observed. Nevertheless, with the advanced medical therapy, it is not uncertainty if a similar circadian pattern of STEMI symptom onset occurs, as well as its possible impact on clinical outcomes. Few long-term data are available. We assess the impact of circadian symptom-onset patterns of STEMI on major adverse cardiovascular events (MACE) in more contemporary patients treated with primary percutaneous coronary intervention (PPCI).

**Methods and results:**

A total of 1099 consecutive STEMI patients undergoing PPCI ≤12h from symptom onset during 2013 to 2019 were classified into 4 groups by 6-h intervals according to time-of-day at symptom onset: night (0:00–5:59), morning (6:00–11:59), afternoon (12:00–17:59), and evening (18:00–23:59). Incidence of MACE including cardiovascular death and nonfatal MI during a median follow-up of 48 months was compared among the 4 groups. A morning peak of symptom onset of STEMI was detected during the period 06:00–11:59 (*p* < .001). Compared with other three 6-h intervals, the incidence of long-term MACE during night onset-time (18.8%, 10.1%, 10.7% and 12.4%, *p* = .020) was significant higher that was driven by more mortality (13.1%, 6.5%, 7.1%and 7.7%, *p* = .044). Night symptom-onset STEMI was independently associated with subsequent MACE (hazard ratio = 1.57, 95%CI: 1.09–2.27, *p* = .017) even after multivariable adjustment.

**Conclusions:**

Circadian variation of STEMI symptom-onset with morning predominance still exists in contemporary practice. Night symptom-onset STEMI was independently associated with increased risk of MACE in Chinese patients treated with PPCI.

## Introduction

Circadian patterns of acute myocardial infarction (AMI) have been known since the 1980s. Following this concept, a number of studies observed the incidence of AMI peaking during sleep to wake transition over the past decades, although a secondary peak in the evening has also been reported [1–3]. However, studies regarding Asian populations are conflicting, either reporting the absence of circadian rhythm or excess between midnight and noon; and limited data exists regarding the circadian onset patterns of AMI in China. Jia et al. reported that the circadian pattern of AMI in Chinese patients showed significant difference from previous studies with three peaks; they postulated that numerous cardiovascular risk markers, both at rest as well as during exercise, could potentially contribute to the day/night pattern for adverse cardiovascular events [4]. While significant advances in the secondary prevention and introduction of primary percutaneous coronary intervention (PPCI), that changed the clinical picture of the acute ST-elevation myocardial infarction (STEMI) population [5], it is uncertainty whether a similar daytime variation of symptom onset of AMI also occurs?

Furthermore, data on whether the time-of-day at symptom onset of AMI affects patients’ prognosis is still conflicting. While some studies presented that mortality differed according to the time of AMI onset, others failed to detect a clear circadian dependence of prognosis after ischaemia [3,6–8]; but, not all patients received PPCI. Little information is available for Asian population on the relevance of the circadian clock of AMI onset in terms of clinical outcomes, especially after implementation of guidelines for the management of patients with STEMI in recent last years.

We, therefore, sought to investigate the time distribution patterns of STEMI symptom onset in a Chinese cohort in contemporary practice; and evaluate the impact of circadian onset patterns of STEMI on the long-term major adverse cardiovascular events (MACE) after PPCI; accordingly obtain useful information for understandings and approaches to AMI patients at increased risk during possible vulnerable periods.

## Methods

This retrospective observational cohort study consisted of all patients (*n* = 1190) who were consecutively admitted for a diagnosis of STEMI and underwent PPCI between January 2013 and September 2019. Acute STEMI was defined as typical increase and decrease of cardiac biomarker values and at least one of the following [9]: (1) symptoms of ischaemia; (2) development of pathologic Q wave in ≥2 contiguous electrocardiogram leads; (3) new significant ST-segment or T- wave change or new-onset left bundle branch block; (4) identification of intracoronary lesion by angiography. Self-reported time of beginning of continuous discomfort compatible with STEMI was defined as symptom-onset time.

**Figure 1.**
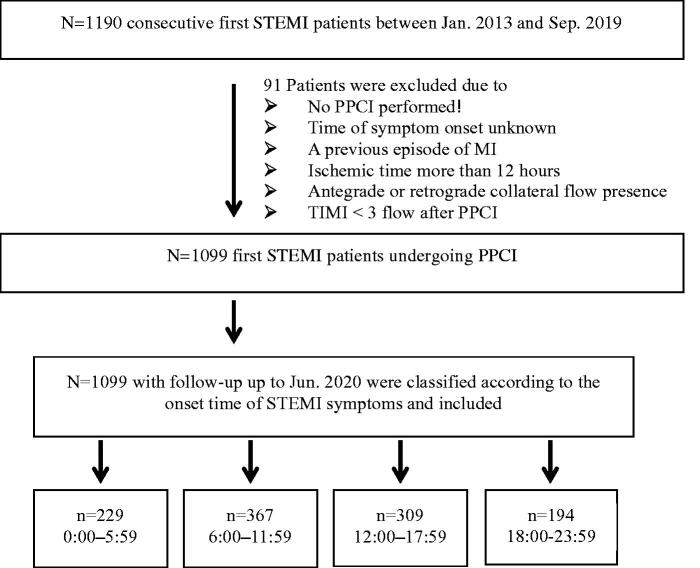
Patient selection flow. *STEMI*: ST-segment elevated myocardial infarction; *PPCI*: primary percutaneous coronary intervention; *TIMI*: Thrombolysis in Myocardial Infarction.

Patient demographic information, medical history, cardiovascular risk factors, laboratory assessments, medical therapy at discharge, and revascularization procedures were collected and recorded in the Cardiovascular Centre Beijing Friendship Hospital Database Bank. Patients were categorized by standard 6-h intervals over 24 h according to the time-of-day at symptom onset: 0:00–5:59 (night, group1), 6:00–11:59 (morning, group2), 12:00–17:59 (afternoon, group 3), and 18:00–23:59 (evening, group4) [3]. As shown in Figure 1, 91 were excluded for (1) no PPCI performed; (2) time of symptom onset unknown; (3) a previous episode of MI; (4) ischaemic time more than 12 h; (5) antegrade or retrograde collateral flow presence; (6) TIMI <3 flow after PPCI. Finally, 1099 patients were included in this analysis. After discharge, all patients were followed up until June 2020.

A composite of MACE including cardiovascular mortality and nonfatal recurrence of MI during follow-up was defined as the primary study end point. Cardiovascular death was defined as death attributed to MI, congestive heart failure, or documented sudden cardiac death. Secondary study end points included cardiovascular death, nonfatal MI separately and repeat PCI that was not due to in-stent thrombosis or as planned procedure. Subsequent MACE after hospital discharge was collected during routine clinical follow-up visits post-STEMI.

All procedures performed in studies involving human participants were in accordance with the Institutional Ethics Committee of the Beijing Friendship Hospital affiliated to Capital Medical University (2020-P2-055-01) and with the 1964 Declaration of Helsinki and its later amendments or comparable ethical standards. This retrospective study was considered minimal risk by the Institutional Ethics Committee; therefore, formal consent is not required.

Statistical analysis was performed using the Statistical Package for Social Sciences, version 24 (IBM Inc, Armonk, NY, USA) software. Continuous variables were described as the mean ± standard deviation or median and interquartile range as appropriate, and categorical variables were expressed as numbers or percentage. Patient characteristics among the 4 onset-time groups were compared by the Kruskal-Wallis test for continuous variables, and by chi-square test or Fisher’s exact test for categorical variables. Spearman’s correlation method was performed to examine the correlation between neutrophil count measured post PPCI and level of peak troponin-I. Cumulative event curves with the Mantel-Haenszel log-rank test were used for comparing MACE over time among the 4 groups.

Multivariate Cox proportional hazards analysis was used to estimate the hazard ratios (HR) and 95% confidence intervals (CI). We included the comparison of time group 00:00 to 05:59 versus all other time groups (06:00–23:59) as a dichotomous variable in the multivariate analysis. Baseline risk factors and co-morbidities (age, sex, body mass index, smoking status, hypertension, diabetes hypercholesterolaemia and prior vascular diseases), clinical presentation characteristics and laboratory parameters (heart rate and systolic blood pressure on admission, anterior location of MI, peak troponin-I, *N*-terminal probrain natriuretic peptide [NT pro-BNP], glycosylated haemoglobin [HbA1c], low-density lipoprotein [LDL] cholesterol), and treatment variables (door to balloon [D2B] time, pre-hospital-delay time, coronary angiography, anti-platelet drugs, angiotensin-converting enzyme inhibitor [ACEI]/angiotensin receptor antagonists [ARBs], βeta-blockers, and statins) were included in the univariate analysis. This multivariate analysis with adjusting for any possible confounding variables identified in the univariate analysis was performed to identify the independent risk predictors.

## Results

Of 1099 patients in this analysis, the mean age of was 62.2 years and 78% were male. As illustrated in Figure 1, more onset of STEMI symptoms occurred in the morning hours (*p* < .001); 229 (20.8%) presented from 0:00 to 5:59, 367 (33.4%) from 6:00 to 11:59, 309 (28.1%) from 12:00 to 17:59, and 194 (17.7%) from 18:00 to 23:59. When stratified by each hour, STEMI symptom onset peaked close to 9 am with a nadir around 11 pm, which corresponds to a 5.0% relatively higher number.

Baseline characteristics were compared among the 4 onset-time groups as shown in Table 1. There were no significant differences in baseline demographic and clinical characteristics with the exception of values of peak CK-MB, peak troponin-I, NT-pro BNP, and left ventricular ejection fraction (LVEF). Angiographic characteristics and the uses of guideline-based medications before discharge were comparable among the 4 onset-time groups. Of note, the utility of chronic preadmission medications was compared, and none of the variables had more than 2% difference from the overall percentage. And a constant increase in the administration of these cardioprotective medications was noted over years (from 34.4% in 2013 to 45.8% in 2019 for statin, from 24% in 2013 to 40.2% in 2019 for aspirin/P2Y12 inhibitor, from 20.4% in 2013 to 29.1% in 2019 for βeta blocker, and from 18.2% in 2013 to 27.4% in 2019 for ACEI/ARB).)

**Table 1.** Comparison of baseline characteristics according to symptom onset-time.

Group variable	0:00–5:59 *N*＝229	6:00–11:59 *N*＝367	12:00–17:59 *N*＝309	18:00–23:59 *N*＝194	*p*-value
Age (years)	63(55, 73)	62(55, 70)	61(53, 70)	60(53, 68)	<.11
Sex (female,%)	50(22)	89(24)	61(20)	42(22)	.57
BMI (kg/m^2^)	25.4 ± 4.2	25.5 ± 4.1	25.4 ± 4.1	25.3 ± 3.3	.94
Heart rate (bpm)	72(64, 82)	72(64, 84)	73(65, 83)	74(65, 83)	.82
SBP (mmHg)	121 ± 23	123 ± 23	123 ± 23	123 ± 22	.70
DBP (mmHg)	71 ± 14	72 ± 14	74 ± 14	74 ± 15	.12
Current smoker (%)	122(53)	188(51)	162(52)	119(61)	.13
Hypertension (%)	138(60)	210(57)	199(64)	107(55)	.14
Diabetes mellitus (%)	63(28)	111(30)	88(28)	52(27)	.82
Hypercholesterolaemia (%)	103(45)	181(49)	154(50)	90(46)	.63
CKD (%)	10(4)	17(5)	13(4)	10(5)	.97
Anterior MI (%)	108(47)	179(49)	154(50)	108(55)	.33
*Medical History*					
CHD (%)	92(40)	141(38)	124(40)	80(41)	.92
CVA (%)	28(12)	40(11)	43(14)	13(7)	.09
PVD (%)	7(3)	15(4)	8(3)	7(4)	.73
*Chronic preadmission medication*					
Aspirin/ P2Y12 inhibitor(%)	89(39)	154(42)	123(40)	76(39)	.89
βeta blocker (%)	67(29)	105(29)	92(30)	56(29)	.98
Statin (%)	104(45)	173(47)	137(45)	89(46)	.93
ACEI/ARB (%)	62(27)	95(26)	88(28)	57(29)	.84
*Coronary artery disease*					
Single-vessel (%)	19(8)	32(9)	30(10)	13(7)	.70
Double-vessel (%)	33(15)	62(17)	48(16)	35(18)	.74
Triple-vessel or LMT (%)	177(77)	273(74)	231(74)	146(75)	.87
CTFC after PCI	26(16–35)	22(14–33)	24(15–36)	24(14–34)	.24
*Laboratory values*					
Peak CK-MB (ng/mL)	207(98–328)	119(50–344)	137(58–256)	165(51–371)	**<.001**
Peak troponin-I (ng/mL)	24(11–50)	15(6–44)	18(7–43)	23(7–50)	**.02**
NT pro-BNP	1876 (858–5155)	1370 (403–3874)	1627 (876–3737)	1715 (642–3483)	**.02**
Haemoglobin A1c (%)	6.3 ± 1.4	6.6 ± 1.6	6.5 ± 1.4	6.2 ± 1.3	.06
Total-C (mmol/L)	4.5 ± 1.1	4.6 ± 1.0	4.5 ± 1.2	4.6 ± 1.0	.36
LDL-C (mmol/L)	2.5 ± 0.8	2.7 ± 0.7	2.6 ± 0.8	2.7 ± 0.7	.15
HDL-C (mmol/L)	1.06 ± 0.25	1.08 ± 0.25	1.07 ± 0.27	1.10 ± 0.26	.27
Triglycerides (mmol/L)	1.5(1.0–2.0)	1.4(1.1–2.0)	1.4(1.0–2.1)	1.4(1.0–2.2)	.78
eGFR (mL/min/1.73m^2^)	84(69–96)	84(71–97)	84(69–97)	88(77–102)	.06
LVEF at discharge (%)	55 ± 10	57 ± 10	57 ± 9	57 ± 9	**.02**
*Medications at discharge*					
Aspirin/ P2Y12 inhibitor(%)	220(97)	350(95)	296(94)	189(98)	.70
β-blocker (%)	184(84)	275(72)	231(75)	154(77)	.29
ACEI/ARB (%)	145(65)	236(63)	201(66)	138(72)	.49
Statin (%)	208(91)	329(88)	274(86)	173(91)	.88

Data are presented as mean ± standard deviation, median (interquartile range) or percentage of patients. Bold values indicate *p* < .05. BMI: body mass index; SBP: systolic blood pressure; DBP: diastolic blood pressure; CKD: chronic kidney disease; MI: myocardial infarction; CHD: coronary heart disease; CVA: cerebrovascular accident; PVD: peripheral vascular disease; ACEI: angiotensin-converting enzyme inhibitor; ARB: angiotensin receptor antagonist; LMT: left main trunk; CTFC: corrected TIMI frame count; PCI: percutaneous coronary intervention; NT pro-BNP: N-terminal probrain natriuretic peptide; C: cholesterol; LDL: low density lipoprotein; HDL: high density lipoprotein; eGFR: estimated glomerular filtration rate; LVEF: left ventricular ejection fraction.

**Figure 2.**
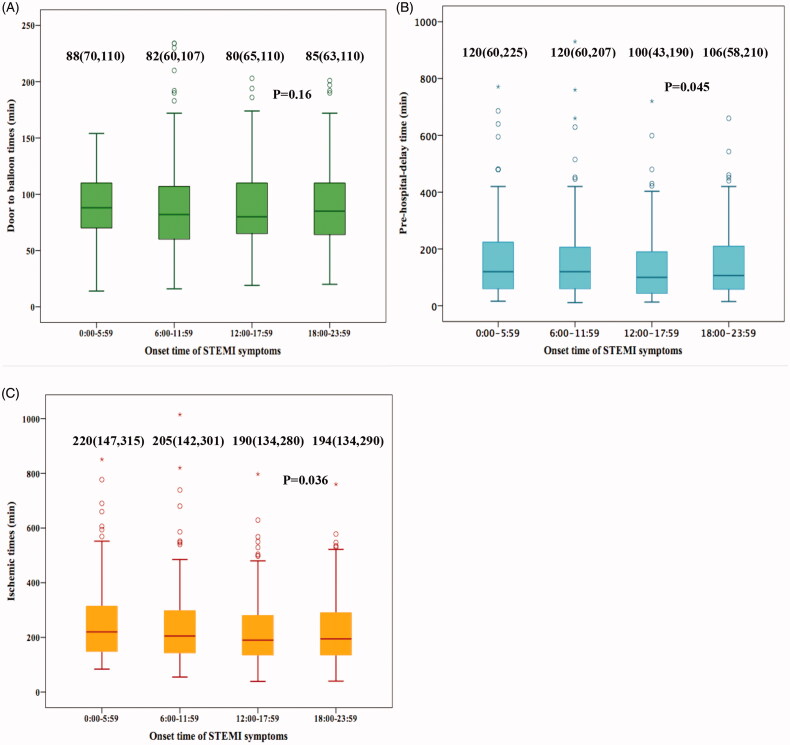
Time variable characteristics. (A–C) The differences (median [IQR]) of door to balloon time, pre-hospital-delay time and ischaemic time among 4 onset times respectively. Top and bottom box edges represent 25th and 75th interquartile range, and error bars represent SD.

Time variable characteristics are shown in Figure 2. No significant differences were noted in D2B time (*p* = .16) (Figure 2(A)). Patients presenting during 0:00–5:59 encountered longer pre-hospital-delay time (*p* = .045) and consequently also longer ischaemic time (*p* = .036) compared to patients within the other onset-time groups (Figure 2(B,C)).

**Figure 3.**
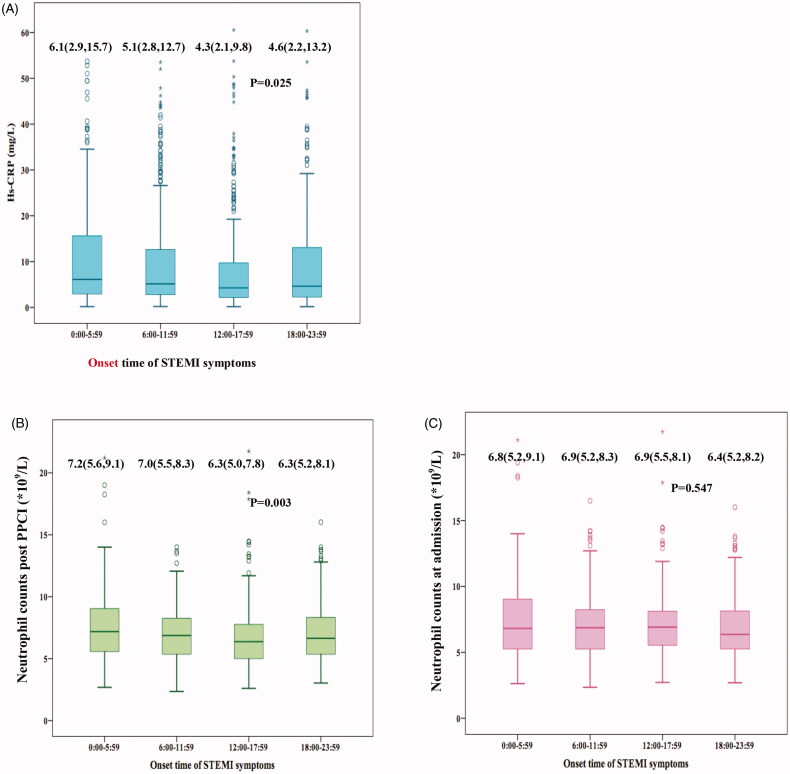
Blood sampling for Hs-CRP and neutrophil count were performed. (A) median level of Hs-CRP measured within 24 h post PPCI in patients with night symptom-onset was significantly higher than other hours of the day (*p* = .025); (B) median level of neutrophil counts measured within 24 h post PPCI in patients with night symptom-onset was significantly higher than other hours of the day (*p* = .003); (C) neutrophil counts at admission were similar among 4 groups (*p* = .547). Top and bottom box edges represent 25th and 75th interquartile range, and error bars represent SD. *Hs-CRP*: high sensitivity C-reactive protein; *PPCI*: primary percutaneous coronary intervention.

Most strikingly, Figure 3 showed that patients with night symptom-onset had significantly higher median levels of Hs-CRP (*p* = .025), neutrophil count (*p* = .003) measured within 24 h post PPCI than those of other hours of the day, while neutrophil count on admission was similar among 4 onset-time groups (*p* = .547). According to correlation analysis, neutrophil count measured post PPCI was associated with the level of peak troponin-I (*r* = 0.612, *p* < .001).

**Figure 4.**
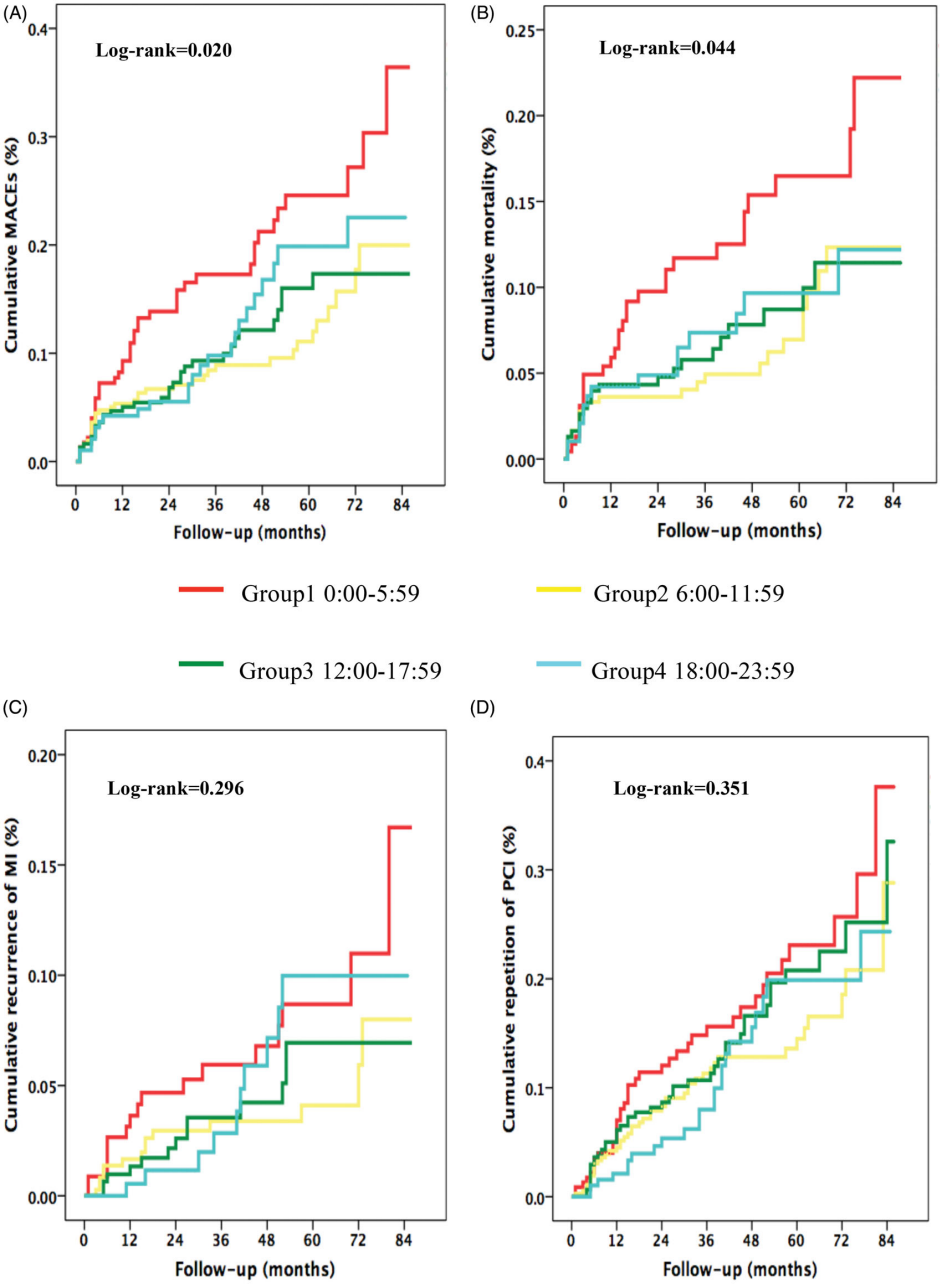
Kaplan–Meier analyses during follow-up according to symptom onset times of STEMI. Cumulative incidence of MACEs in panel (A) cardiovascular mortality in panel. (B) recurrence of MI in panel (C) and repeat PCI in panel (D) *MACE*: major adverse cardiac events; *MI*: myocardial infarction; *PCI*: percutaneous coronary intervention.

Among 1099 patients in this analysis, 97% completed the clinical follow-up with a median duration of 48 months. A total of cumulative incidence of MACE was 12.5% (*n* = 137) during the follow-up. Cardiovascular death occurred in 91 cases, nonfatal MI in 54 patients, and 149 unplanned repeat PCI. The Kaplan–Meier event curves (Figure 4) showed that incidence of long-term MACE was significantly higher during night onset-time compared with the rest of the day (18.8%, 10.1%, 10.7% and 12.4%, *p* = .020 by log-rank test). There was a significant difference in mortality among the 4 groups (*p* = .044 by log-rank test), with night symptom-onset group being the highest (13.1%), followed by evening group (7.7%), afternoon group (7.1%) and morning group (6.5%), respectively. The secondary endpoint comparisons showed nonfatal MI rates (7.4%, 3.8%, 4.2% and 5.2%, *p* = .296 by log-rank test) among the 4 onset-time groups, and unplanned repeat PCI (17%, 12%, 14% and 12%, *p* = .351 by log-rank test).

Univariable Cox regression analysis identified a list of covariates mentioned earlier that were significantly associated with risk of subsequent MACE in STEMI patients underdoing PPCI as shown in Table 2. The relative risk of MACE during night onset-time was 1.84 compared with other hours of the day (95% CI 1.22–2.78, *p* = .004). In the multivariate analysis, this result did not generally change after stratification with potential confounding variables. Night symptom-onset STEMI was significantly and independently associated with risk of MACE (HR = 1.57, 95%CI: 1.09–2.27, *p* = .017). In addition, levels of peak troponin-I, NT pro-BNP, Triple-vessel or LMT lesions, and use of aspirin were independent associated with risk of MACE (Table 2).

**Table 2.** Cox Proportional Hazards Regression Analyses for MACE.

	Univariate	Regression		Multivariate	Regression	
Variable	HR	95%CI	*p* value	HR	95%CI	*p* value
Night symptom-onset time	1.84	1.22–2.78	**.004**	1.57	1.09–2.27	**.017**
Age	1.31	0.91–1.88	.14			
Women	1.11	0.87–1.42	.41			
BMI	1.01	0.99–1.02	.77			
Current smoker	0.81	0.52–1.27	.36			
Heart rate	0.99	0.79–1.24	.97			
SBP	1.00	0.99––1.01	.50			
Anterior MI	1.11	0.79–1.55	.53			
Hypertension	0.99	0.98–1.00	.33			
Diabetes mellitus	1.05	0.71–1.56	.80			
Hypercholesterolaemia	1.17	0.83–1.64	.36			
Prior CHD	0.98	0.78–1.23	.90			
Peak troponin-I	1.01	1.00–1.02	**.022**	1.04	1.03–1.05	**<.001**
Haemoglobin A1c	0.99	0.98–1.00	.37			
LDL cholesterol	0.82	0.65–1.03	.09			
NT pro-BNP	1.00	1.00–1.00	**<.001**	1.00	1.00–1.00	**<.001**
Pre-hospital-delay time	1.00	1.00–1.00	**.018**	1.00	1.00–1.00	.09
Door to balloon time	1.00	0.99–1.01	.36			
Triple-vessel or LMT lesions	1.87	1.27–2.76	**.001**	2.08	1.18–3.65	**.011**
Aspirin	3.27	2.67–4.01	**<.001**	2.53	1.63–3.95	**<.001**
ACEI/ARB	1.57	1.33–1.86	**<.001**	1.16	0.95–1.42	.13
Statin	1.84	1.52–2.22	**<.001**	0.90	0.61–1.31	.60
βeta-blocker	1.36	1.14–1.62	**<.001**	0.95	0.74–1.22	.72

Variables (*p* < .05) of clinical outcome identified through univariate analysis were tested in a multivariate analysis. Bold values indicate *p* < .05. BMI: body mass index; SBP: systolic blood pressure; MI: myocardial infarction; CHD: coronary heart disease; LDL: low density lipoprotein; NT pro-BNP: N-terminal probrain natriuretic peptide; LMT: left main trunk; ACEI: angiotensin-converting enzyme inhibitor; ARB: angiotensin receptor antagonist; MACE: major adverse cardiac events.

## Discussion

We present here results that enabled us to gain a comprehensive insight regarding real-world circadian distribution of STEMI according to symptom onset in contemporary practice, and the impact of circadian onset patterns of STEMI on the risk of MACE during long-term follow-up in a cohort of more contemporary patients who underwent PPCI. We found that: (1) despite the advanced medical therapy, a circadian rhythm of STEMI still exists. The onset of STEMI symptoms occurred more frequently in the morning. (2)follow-up MACE occurred significantly more in patients during night onset-time; (3)night symptom-onset STEMI was independently associated with long-term MACE (HR = 1.57, 95%CI: 1.09–2.27, *p* = .017) even after multivariable adjustment; and (4) patients with night symptom-onset had significantly higher median levels of Hs-CRP and neutrophil count measured within 24 h post PPCI than those of other hours of the day.

Our findings not only corroborate previous pilot studies, which showed a peak in symptom onset of AMI during the dark-to-light transition period [1–3], but also demonstrated that despite major advances in medical therapy over time, the effects of circadian rhythm on AMI occurrence have remained constant. As we known, since the first report of circadian patterns of AMI, significantly changed lifestyles and improvements in medical care have been made. The present study showed that, accompany with the increased frequency of hypertension and hypercholesterolaemia, the increased utility of cardioprotective pharmaceuticals has also been observed over time. Pharmacotherapy with these proven cardiovascular agents may exert their effects partially because they protect against triggered events [10,11].

Possible explanations include the critical influence of circadian rhythms on AMI pathophysiology that can create a circadian-regulated window of vulnerability that underlies ischaemia. Circadian regulated factors exhibit a pro-thrombotic tendency and/or pro-ischaemic state in the early daytime hours [10,11]. However, most importantly, coupled with this underlying pathophysiology that generates a vulnerable substrate, a patient’s background and physiological circadian rhythms might complexly interact with each other; and the presence of clock genes has been reported in human cardiac tissue that displays a significant time-dependent transcriptional variation coinciding with myocardial events [12].

The relationship between AMI onset-time and the clinical outcomes is controversial [2,3,6–8,13]. A meta-analysis including 30 studies on AMI and 19 studies on sudden cardiac death showed that more than 27% of morning AMIs and more than 22% of sudden cardiac deaths were attributable to a morning excess of risk [13]. While Fournier et al. reported that patients with a night-onset AMI (between 00:00 and 05:59) had the worst 30-day mortality [3]. On the other hand, Holmes et al. [2] observed no significant association between the circadian patterns of onset time and in-hospital mortality in patients with STEMI after adjusting for clinical risk factors. Most previous studies have analysed short-term in-hospital, 30-day or 1-year mortality. Recently, Sager and colleagues [14] reported that time-of-day at symptom onset was neither associated with infarct size nor with 5-year mortality in patients with STEMI undergoing PPCI. However, in their study, STEMI patients enrolled between 2002 and 2007 were not be the most contemporary STEMI cohort with respect to stent technology and anti-platelet therapy; and only 37% patients finished 5-year follow-up. But our study represents the analysis of an association between circadian onset patterns of STEMI and long-term clinical outcomes in more contemporary patients who underwent PPCI. Our findings showed that although more onsets of STEMI symptoms occurred in the morning hours, patients with night symptom-onset had higher incidence of adjusted follow-up MACE and long-term mortality. A night symptom-onset STEMI was independently associated with the increased risk of long-term MACE.

Previous studies also have described an “off-hours effect” that AMI patients admitted to hospital during off-hours experience poorer clinical outcomes [15,16]. Several studies have suggested that the higher mortality rate during off-hours could be partially explained by the lower implementation rate of PCI [15,17], but all of our patients received PPCI implementation. And final achievement of TIMI 3 flow, as well as the corrected TIMI frame count (CTFC) after PPCI as measures of epicardial and microvascular patency did not differ according to the time of day, suggesting the quality of treatment with PPCI is equal during normal duty hours and off duty hours. A meta-analysis [18] reported that patients admitted during off-hours were less likely to receive PCI within 90 min, but this may have a relatively small effect on our analysis due to the comparable door-to-balloon time among the 4 groups; and after adjusting for clinical risk factors, pre-hospital-delay time did not significantly affect the follow-up MACE.

It is worth noting that reperfusion post-MI also triggers an “ischemia/reperfusion (I/R) injury” which exacerbates cardiac remodelling and worsen outcome [19]. A common feature of I/R injury is an inflammatory reaction, with infiltration of polymorphonuclear leukocytes, predominantly neutrophils that have been causally linked to myocardial damage during I/R injury [20,21]. To gain further insight into the I/R injury, we investigated the levels of inflammatory markers after PPCI. Interestingly, in our study population, CRP, an acute phase inflammatory marker, as well as neutrophil levels post reperfusion in patients with night symptom-onset were higher as compared to those of other hours of the day. It suggests that reperfusion elevates infiltration of neutrophils and might translate into adverse remodelling and decreased cardiac function beyond the initial ischaemic injury. This is reflected by our data that neutrophil count measured post PPCI was associated with the level of peak troponin-I, which translated into the difference of LVEF at discharge. STEMI patients with night symptom-onset might have a higher cardiomyocyte vulnerability to I/R injury, which regulated by cardiomyocyte circadian clock genes has been suggested [22,23].

## Limitations

Our study has some limitations. First, the exact time of onset of symptoms is sometimes hard to determine because the information is subjective. Pre-infarction angina may also have occurred and contributed further to an imprecise determination of STEMI onset. However, adjustment for pre-hospital-delay time did not impact the investigated outcomes. Second, this is a single-centre study with a relatively small sample size, but our STEMI system of care allowed us to obtain 4 homogeneous groups in terms of clinical characteristics and management. Finally, we did not have access to the adherence of cardiovascular medication during the follow-up that might have had an impact on clinical outcomes. Although undesirable, we do not believe that these limitations impact on the main findings of the study.

## Conclusions

The present study shows that despite the optimisation of medical therapy for cardiovascular disease over time, circadian pattern of onset time of STEMI symptoms with a morning peak in Chinese population still exists. Night symptom-onset STEMI was independently associated with increased risk of long-term MACE in more contemporary patients treated with PPCI. These results provide interesting data for a better daily planning of the management of STEMI when assessing the prognosis.

## Data Availability

The data that support the findings of this study are available from Cardiovascular Centre Beijing Friendship Hospital Database Bank. But restrictions apply to the availability of these data, as this health information is considered sensitive information under the Privacy Act, hence they are not publicly available.
